# Diabetic nephropathy with marked extra-capillary cell proliferation: a case report

**DOI:** 10.1186/s12882-023-03204-3

**Published:** 2023-05-22

**Authors:** Madoka Morimoto, Tomoko Namba-Hamano, Shoki Notsu, Yukimasa Iwata, Yumiko Yasuhara, Masafumi Yamato, Yoshitaka Isaka

**Affiliations:** 1 Department of Nephrology, Sakai City Medical Center, 1-1-1 Ebaraji-Cho, Nishi-Ku, Sakai City, Osaka Japan; 2 Department of Nephrology, Osaka University Graduate School of Medicine, Osaka, Japan; 3 Department of Pathology, Sakai City Medical Center, Sakai, Japan

**Keywords:** Diabetic nephropathy, Extra-capillary cell proliferation, Crescentic glomerulonephritis, Focal segmental glomerulosclerosis, Podocyte, Parietal epithelial cell

## Abstract

**Background:**

Extra-capillary hypercellularity is a common finding in crescentic glomerulonephritis (GN) and focal segmental glomerulosclerosis (FSGS). In diabetic nephropathy (DN), extra-capillary hypercellularity is often observed as a finding of complications such as IgA nephropathy or microscopic polyangiitis superimposed on DN. However, in rare cases, epithelial cell proliferation may accompany DN. We experienced a case of nodular diabetic glomerulosclerosis with marked extra-capillary hypercellularity and revealed the origin of this atypical lesion using immunostainings.

**Case presentation:**

A man in his 50 s was admitted to the hospital with nephrotic syndrome, and a renal biopsy was performed. Diffuse nodular lesions and extra-capillary hypercellularity were observed, but the results of serological examination or immunofluorescent assays did not implicate any other crescentic GN. Immunostaining for claudin-1 and nephrin was performed to identify the origin of the extra-capillary lesions. Given the clinical course and pathological findings, a diagnosis of DN-associated extra-capillary cell proliferation was made.

**Conclusions:**

Extra-capillary hypercellularity, which resembles FSGS or crescentic GN, is a rare finding in DN and should therefore be treated with caution. In such cases, co-staining for claudin-1 and nephrin may facilitate the diagnosis of DN.

**Supplementary Information:**

The online version contains supplementary material available at 10.1186/s12882-023-03204-3.

## Background

Diabetic nephropathy (DN) is the most common cause of end-stage renal failure in developed countries [1]. Glomerular involvement in DN begins with the thickening of the basement membrane and progresses to a diffuse increase in the mesangial matrix and nodular lesions [2]. When the clinical presentation is consistent with DN, a kidney biopsy is rarely performed, and the diagnosis is made clinically. Biopsies are mainly performed when other renal complications are suspected.

We experienced an atypical case of DN with marked extra-capillary cell proliferation. In this report, we examined the origin of extra-capillary hypercellularity using immunostainings and compared this case with other glomerular diseases with extra-capillary cell proliferation.

## Case presentation

The patient was a male in his 50 s who presented with a chief complaint of dyspnea and edema of the lower legs.

One year prior to admission, the patient was diagnosed with type 2 diabetes mellitus and hypertension. The patient was then observed without medication. Two months before admission, gradually worsening edema of the lower legs appeared, accompanied by dyspnea. The patient was then admitted to another hospital. Nephrotic syndrome and bilateral proliferative retinopathy were found, and the patient was transferred to our hospital approximately one week later.

On admission, a physical examination revealed a weight gain of approximately 30 kg, hypertension of 160/90 mmHg, and SpO_2_ of 98% with 1 L of oxygen inhalation. Respiratory sounds were diminished in both chests, and a Levine II systolic murmur was heard at the third intercostal space of the left sternum border. The abdomen was distended. Edema and stasis dermatitis were observed in both lower legs.

The laboratory findings on admission are shown in Table 1. The patient had nephrotic syndrome with a serum albumin level of 1.5 g/dl and urinary protein excretion of 9.21 g/gCr, accompanied by microscopic hematuria and renal impairment with an estimated glomerular filtration rate (eGFR) of 15.81 ml/min/1.73 m^2^. Mild elevation of immunoglobulin A (IgA) and C-reactive protein levels were noted, but antinuclear antibody, anti-neutrophil cytoplasmic antibody (ANCA), anti-glomerular basement membrane (GBM) antibody, and cryoglobulin were negative. Chest radiography revealed bilateral pleural effusion. Both kidneys were normal in size on computed tomography.

**Table 1:** The laboratory findings on admission

Urinalysis		Blood cell count	
Protein	3 +	WBC	8460 /μl
Occult blood	3 +	Hemoglobin	7.8 g /dl
RBC	20–29 /HPF	MCV	90.7 fl
Granular casts	1 +	Platelet	26.6 × 10^4^ /μl
Protein/Cr	9.21 g/gCr		
Blood chemistry		Serology	
AST	15 U/l	C3	112.5 mg/dl
ALT	9 U/l	C4	31.8 mg/dl
LDH	306 U/l	IgG	1358 mg/dl
Total protein	5.2 g/dl	IgA	528 mg/dl
Albumin	1.5 g/dl	IgM	67 mg/dl
BUN	49.2 mg/dl	ANA	(-)
creatinine	3..41 mg/dl	anti-GBM antibody	(-)
Blood glucose	189 mg/dl	MPO-ANCA	(-)
HbA1c	7.4%	PR3-ANCA	(-)
Total cholesterol	152 mg/dl	ASO	68 U/ml
CRP	1.62 mg/dl	cryoglogulin	(-)

*Abbreviations*: *RBC* Red blood cells, *WBC* White blood cells, *HPF* High power field, *AST* Aspartate aminotransferase, *ALT* Alanine aminotransferase, *LDH* Lactate dehydrogenase, *BUN* Blood urea nitrogen, *CRP* C reactive protein, *ANA* Anti-nuclear antibody, *ANCA* Anti-neutrophil cytoplasmic antibody, *ASO* anti-streptolysin O antibody

**Fig. 1:**
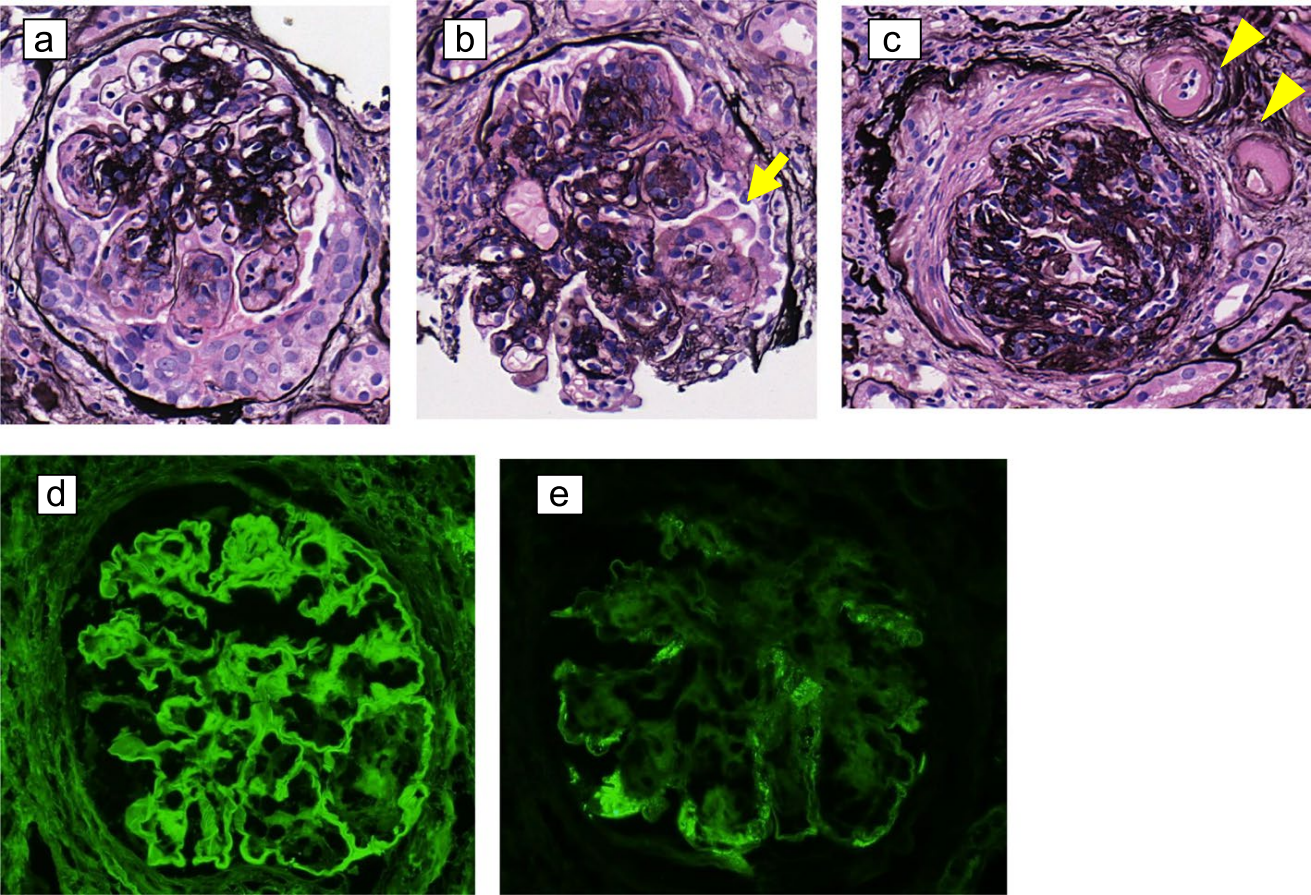
Pathological findings on light microscopy and immunofluorescence staining in this case (**a**) Extra-capillary cell proliferation. **b** A nodular lesion, with some podocyte detachment (arrows). **c** The collapsed glomeruli with a fibrous crescent-like lesion and severe hyalinosis of the arterioles (arrowheads) (**a**, **b** and **c**: Periodic acid-methenamine silver staining, original magnification, 400 ×). **d** Immunofluorescence staining for IgG was linearly positive along GBM (400 ×). **e** Immunofluorescence staining for IgA was positive in peripheral capillary loop (400 ×). IgG, immunoglobulin G; GBM, glomerular basement membrane; IgA, immunoglobulin A

**Fig. 2:**
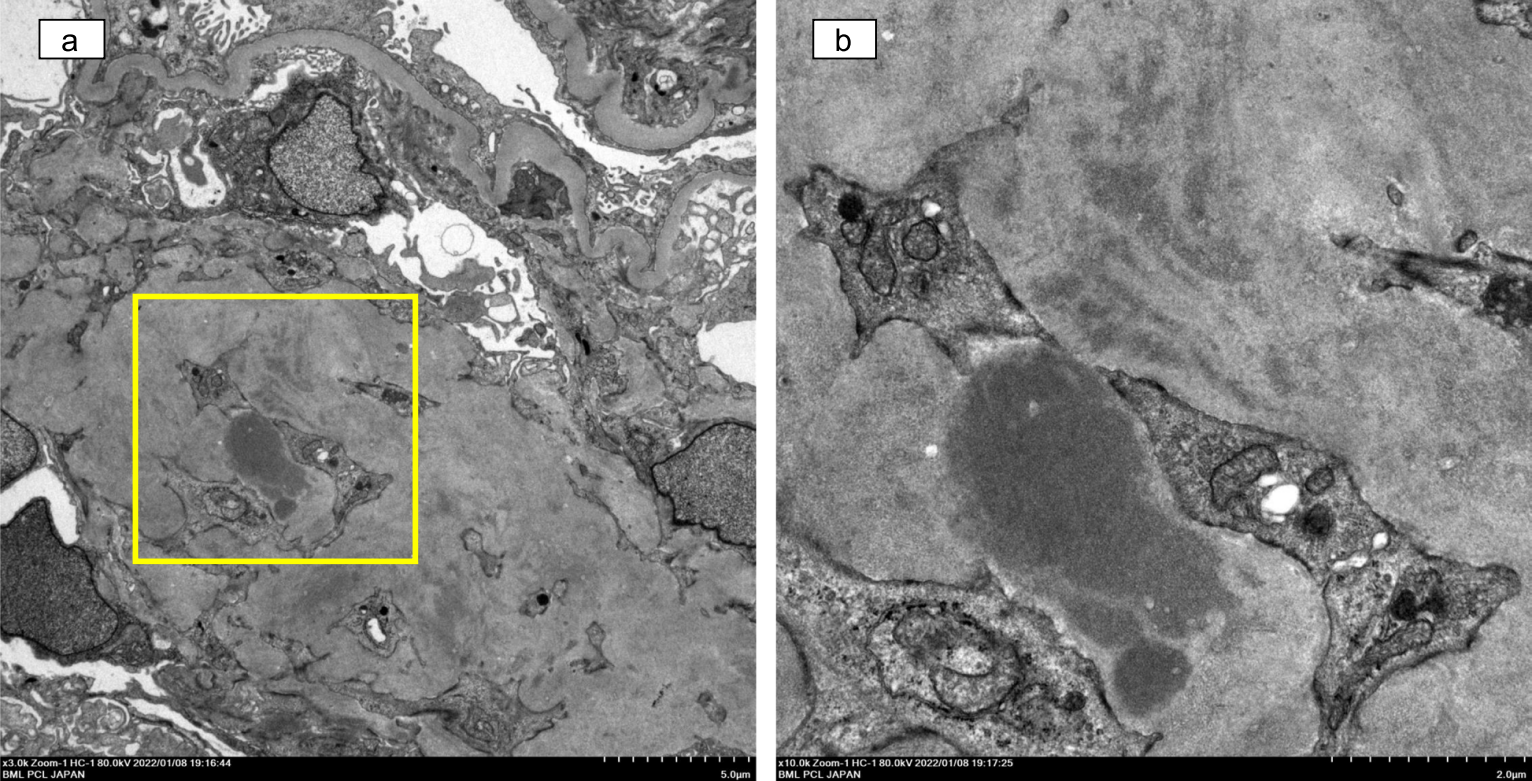
Transmission electron microscopic findings of this case. **a** Expansion of extracellular matrix in the mesangial area, diffusely thickened basement membrane, and foot process effacement along capillary tuft (original magnification, 3000 ×). **b** Magnified view of the framed area in (**a**). Electron-dense material in the mesangial area (original magnification, 10,000 ×)

Ultrasonography-guided percutaneous renal biopsy was performed after diuresis with loop diuretics and tolvaptan. On light microscopy, 42 glomeruli were observed, 6 of which were globally sclerotic. The glomeruli were diffusely enlarged and showed mesangial matrix expansion and nodular lesions (Fig. 1a and b). Twenty-three glomeruli revealed extra-capillary hypercellularity, as shown in Fig. 1a, b, and c. Some lesions were accompanied by podocyte detachment (Fig. 1b). The interstitial fibrosis and tubular atrophy were up to 20%, and focal mild lymphocyte infiltration was seen. Hyalinosis of arterioles (Fig. 1c) and severe arteriosclerotic changes in the interlobular arteries were observed. Immunofluorescence analysis revealed positive staining for immunoglobulin G (IgG) along the GBM (Fig. 1d). IgA, IgM, C4, and C1q were positive in the capillary loop as exudative lesions (Fig. 1e). Transmission electron microscopy (TEM) showed that 80% of the foot processes were effaced, and the GBM was markedly thickened with a dense layer of up to 800 nm (Fig. 2a). Mesangial matrix expansion and electron-dense material were observed in the mesangial area. As the contour of the electron-dense material was indistinct, this material was thought to be derived from an exudative lesion (Fig. 2b).

**Fig. 3:**
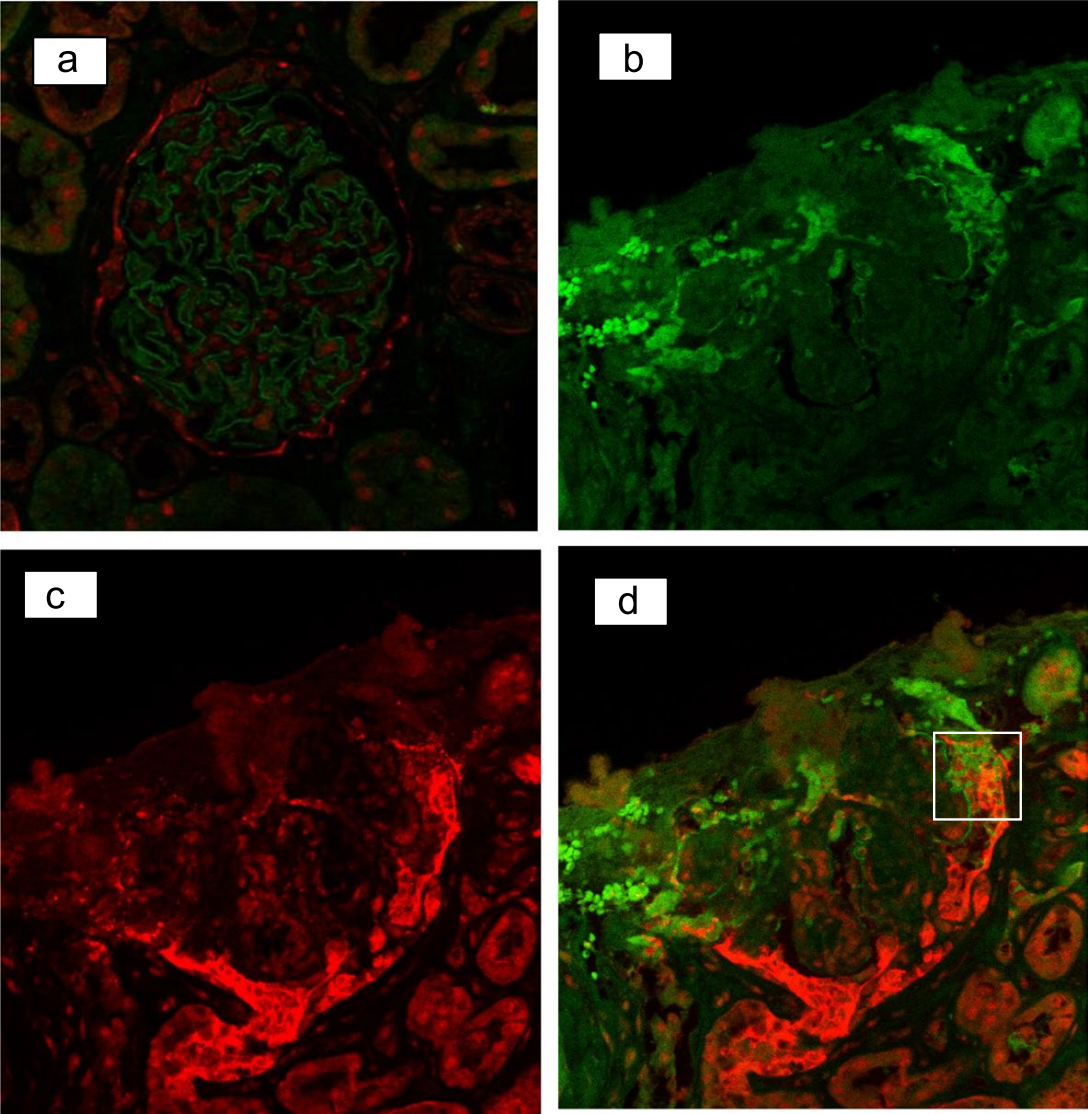
Immunostaining for nephrin(green) and claudin-1(red), in normal control and extra-capillary cell proliferation in this case. **a** Nephrin was expressed in podocytes and claudin-1 was expressed in PECs in normal control (0 h biopsy of transplant graft). **b** Nephrin was down-regulated in the capillary loop. **c** Claudin-1 was strongly positive in extra-capillary cell proliferations. **d** Merged (**b**) and (**c**). Nephrin and claudin-1 was co-stained in the area surrounded by the lines

Immunostaining with anti-claudin-1 and anti-nephrin antibodies was performed to clarify the origin of cells forming the extra-capillary lesions. Immunostaining methods are detailed in supplemental data (Additional file 1). In the normal control (0 h biopsy of transplant graft), nephrin was expressed in podocytes along the capillary loop, and claudin-1 expression was consistent with parietal epithelial cells (PECs) on the Bowman's capsule (Fig. 3a). In the present case, expression of nephrin in podocytes was segmentally decreased, and the extra-capillary hyperplastic areas were mainly positive for claudin-1, with some co-staining of nephrin and claudin-1 (Fig. 3b, c, and d).

**Fig. 4:**
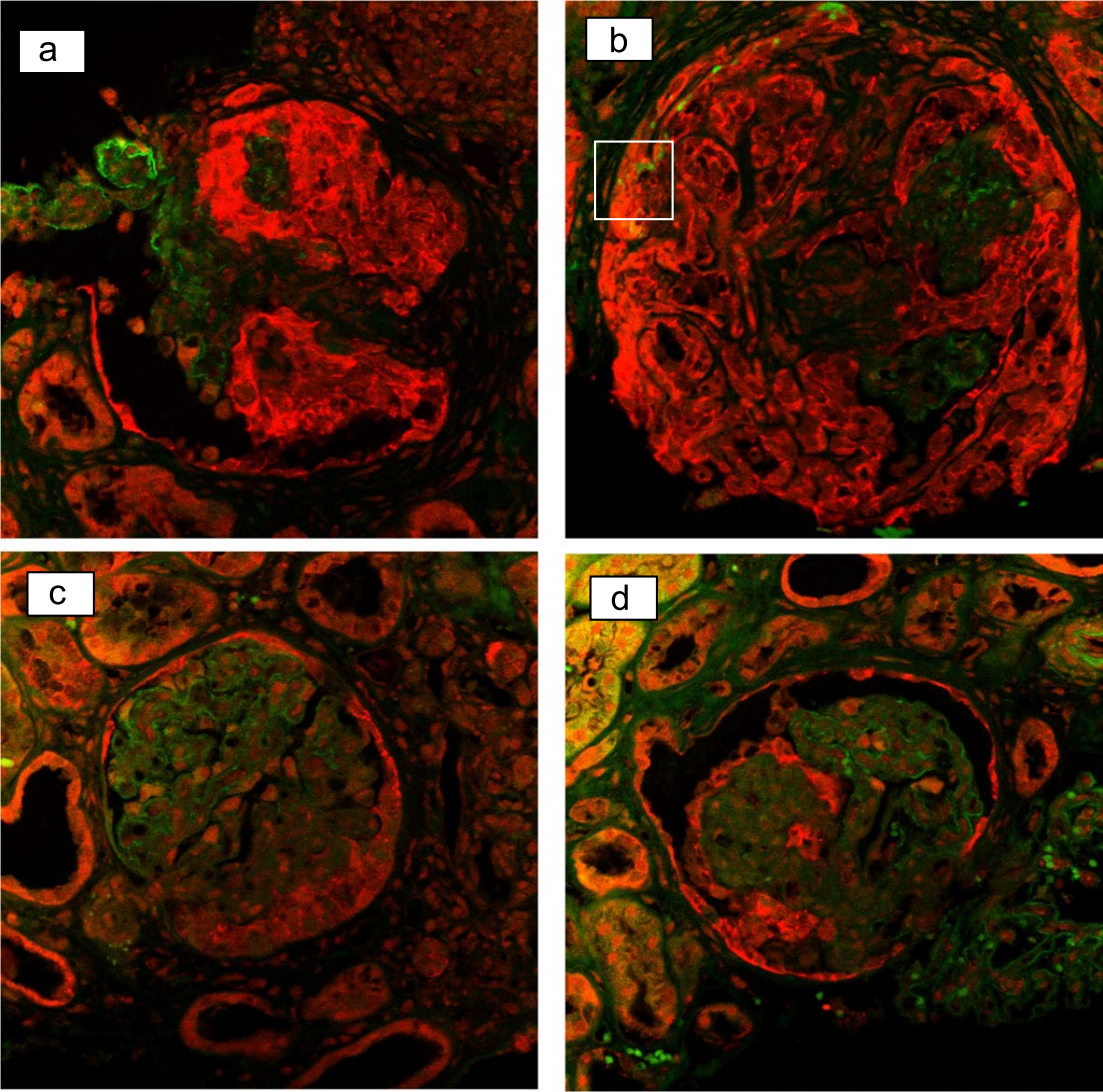
Immunostaining for nephrin (green) and claudin-1 (red) in the extra-capillary proliferation in other diseases. **a** In a case with MPA, claudin-1 was strongly positive in the extra-capillary proliferation and nephrin was positive in the remaining podocytes. **b** In a case with anti-GBM-Ab GN superimposed on DN, the same pattern as MPA was observed with less residual capillary loop than MPA. Co-staining of nephrin and claudin-1 was suspected in the framed area. **c** In a case with FSGS collapsing variant, normal capillary loop expressing nephrin, and extra-capillary epithelial cells expressing claudin-1 were observed. **d** In a case with FSGS NOS variant, almost the same pattern as the collapsing variant was seen. Claudin-1 was expressed in epithelial cells adjacent to sclerotic lesions. MPA, microscopic polyangiitis; GBM, glomerular basement membrane; Ab, antibody; GN, glomerulonephritis; DN, diabetic nephropathy; FSGS, focal segmental glomerulosclerosis

For comparison, the same staining was performed for microscopic polyangiitis (MPA), anti-GBM antibody glomerulonephritis (GN) superimposed on DN, focal segmental glomerulosclerosis (FSGS) collapsing variant, and FSGS NOS (not otherwise specified) variant (Fig. 4a-d). In MPA and anti-GBM antibody GN, nephrin was expressed in podocytes along the capillary loop, similar to the normal control and claudin-1 in extra-capillary lesions. In the two cases of FSGS, nephrin expression in sclerotic lesions decreased, and proliferating epithelial cells were positive for claudin-1.

Based on the co-staining of nephrin and claudin-1, the extra-capillary lesion of this case was likely to be derived from DN.

Given the marked extra-capillary hypercellularity on the light microscopy and no significant deposition by immunofluorescent assay, the patient was initially treated with 30 mg/day (0.5 mg/kg) of oral prednisolone with suspicion of seronegative crescentic GN superimposed on DN. Subsequently, based on TEM and immunostaining findings, we made a diagnosis of diabetes-related extra-capillary cell proliferation, and prednisolone was quickly tapered. RAS inhibitors and SGLT2 inhibitors were added as a treatment for DN. At present, 10 months after the biopsy, the patient maintains an eGFR of 24.44 ml/min/1.73 m^2^ and urinary protein of 1.61 g/gCr, which indicates incomplete remission type II for nephrotic syndrome.

## Discussion and conclusions

In the past report, renal biopsy in 273 patients with type 2 diabetes mellitus revealed DN in 24.9%, non-diabetic renal disease in 64.1%, and mixed type in 11.0%. The histological types of non-diabetic renal diseases vary widely [3].

In this case, we initially thought the patient had crescentic GN superimposed on DN because of the marked extra-capillary hypercellularity. There are several reports of DN complicated by crescentic GN, such as ANCA-associated nephritis [4, 5]. On the other hand, there are also reports of DN with crescent formation [4, 6]. Elfenbein et al. reported the rate of crescent formation in cases of renal biopsy or autopsy-confirmed DN. They described that 25.3% of biopsied cases and 17.98% of autopsy cases had crescents and that the rate of crescents was associated with blood urea nitrogen and serum creatinine levels [6]. Otani et al. reported a case with a clinical background and pathologic features similar to our case. They made a diagnosis of crescentic DN and did not administer immunosuppressive treatment [7].

Salvatore et al. retrospectively examined 534 patients with diabetes mellitus who were biopsied for increased urinary protein levels or worsened renal function [8]. The extra-capillary cell proliferation observed in DN was reported to be similar to a pseudo-crescent observed in collapsing glomerulopathy (CG), and 26 (5%) patients had CG findings in addition to DN. Patients with CG findings often had advanced DN, and severe arteriosclerotic findings. Therefore, they hypothesized that ischemia may trigger CG onset in DN.

Mottl et al. examined the pathology associated with renal prognosis in 109 biopsied cases with sole findings of diabetic nephrosclerosis [9]. They reported segmental sclerosis and extra-capillary hypercellularity as poor prognostic factors for end-stage renal failure. They also described that extra-capillary hypercellularity in DN resembles that of CG rather than crescentic GN.

Gaut et al. examined cellular crescents in diabetic glomerulosclerosis by staining for claudin-1 and nephrin and compared them to inflammatory crescents [10]. Claudin-1 is a transmembrane protein expressed at the tight junctions of PEC, and nephrin is a slit membrane protein in podocytes. They reported that diabetic glomeruli with crescents had cells that co-expressed claudin-1 and nephrin. Co-stained cells were not observed in the inflammatory crescent. These findings were similar to those in the present case.

Andeen et al. examined podocyte and PEC markers in DN and reported that claudin-1-positive cells increase in the tuft as DN progressed, which leads to "capping" in the sclerotic region [11]. They also reported cells co-expressing podocyte markers p57 and claudin-1. They hypothesized that podocyte progenitor cells in the PECs or Bowman's capsule migrate to the tuft as compensation for podocyte loss.

It is still controversial whether the origin of extra-capillary cell proliferation in FSGS is from the podocytes or PECs [12–15]. In our study, we examined two cases of FSGS: the collapsing and NOS variant. Both showed decreased nephrin staining in sclerotic lesions, and proliferating epithelial cells were positive for claudin-1. Although not observed in our study, there are several reports of podocyte and PEC markers co-staining in segmental sclerotic lesions [15, 16].

Podocyte injury and loss have been reported to be common findings in both FSGS and DN indicating glomerular injury [17, 18]. We hypothesize that DN and FSGS share similar pathological and immunohistochemical features due to sharing common pathogenesis.

On the other hand, extra-capillary hypercellularity in crescentic GN is also positive for PEC markers [8, 19, 20]. However, co-staining for podocyte and PEC markers has not been reported.

In this report, we examined a patient with anti-GBM antibody GN. The patient presented as a representative example of anti-GBM antibody GN had a history of diabetes mellitus for approximately 2 years and early DN. Its pathological findings were classified as Renal Pathology Society (RPS) classification class I, with thickening of the GBM, as revealed by electron microscopy. Some extra-capillary hypercellularity lesions were suspected to be co-stained with claudin-1 and nephrin (Fig. 4b). It would be difficult to determine whether the extra-capillary hypercellularity was caused by the anti-GBM antibody GN or DN. Previous reports have shown that the duration of diabetes mellitus in DN with extra-capillary hypercellularity ranges from 0 to 18 years [21]. In contrast, glomerular involvement in DN with extra-capillary hypercellularity is seen in patients with RPS classification class IIa or higher. The more advanced the glomerular lesion, the more often extra-capillary hyperplasia is present. In our GBM antibody GN case, the major findings were crescentic GN with 90% of glomeruli having crescent, whereas findings of DN were mild as class I according to the RPS classification. Therefore, the extra-capillary hypercellularity in this case may be a crescentic GN.

The limitations of this study include the fact that this was a single case. In addition, the involvement of CD44-positive activated PECs and renal progenitor cells has recently been investigated in diseases involving extra-capillary cell proliferation [19, 22]; however, the involvement of these cells was not examined in this study.

In conclusion, although DN with extra-capillary hypercellularity is a rare condition, investigation of extra-capillary lesions using immunostainings may be meaningful.

## Supplementary Information

**Additional file 1.** Concise methods. Brief overview of immunostaining methods and antibodies used.

## Data Availability

Data sharing is not applicable to this article as no datasets were generated or analyzed during the current study.

## Abbreviations

ANCA: Anti-neutrophil cytoplasmic antibody
CG: Collapsing glomerulopathy
DN: Diabetic nephropathy
eGFR: Estimated glomerular filtration rate
FSGS: Focal segmental glomerulosclerosis
GBM: Glomerular basement membrane
GN: Glomerulonephritis
IgA: Immunoglobulin A
IgG: Immunoglobulin G
MPA: Microscopic polyangiitis
NOS: Not otherwise specified
PEC: Parietal epithelial cells
PBS: Phosphate-buffered saline
RPS: Renal pathology society
TEM: Transmission electron microscopy
