# Supplementary material for: Diabetic nephropathy with marked extra-capillary cell proliferation: a case report
Source: BMC Nephrol. 2023 May 22;24:139. doi: 10.1186/s12882-023-03204-3 (PMC10201740; doi:10.1186/s12882-023-03204-3)
Supplement: Supplementary file 1 — Additional file 1. Concise methods. Brief overview of immunostaining methods and antibodies used. [file 12882_2023_3204_MOESM1_ESM.docx]

# **ADDITIONAL FILES**

File name:　Additional file 1

File format: .docx

Title of data: Concise methods.

Description of data: Brief overview of immunostaining methods and antibodies used.

# **CONCISE METHODS**

**Immunostaining Methods**

Paraffin sections of 3-μm thickness were deparaffinized, immersed in 0.01 mM citric acid buffer (pH 6), and autoclaved at 120 °C for 10 min for antigen activation. Then, blocking was performed with 1% bovine serum albumin for 60 min at room temperature, and the primary antibody was incubated at 4 °C overnight. After washing three times with phosphate-buffered saline (PBS), the secondary antibody was reacted for 30 min at room temperature, washed three times with PBS, and sealed using VECTASHIELD Mounting Medium (H-1000; Vector Laboratories). The tissue sections were observed and photographed using an Olympus FV1000-D confocal laser scanning microscope.

**Antibodies Used**

The primary antibodies used were GP-N2 from PROGEN, diluted 200-fold for anti-nephrin antibodies, and ab15098 from Abcam, diluted 200-fold for anti-Claudin-1 antibodies.

The secondary antibodies used were Alexa Fluor™ 488 Goat anti-Guinea Pig IgG (H+L) Highly Cross-Adsorbed Secondary Antibody (A11073; Thermo Fisher Scientific) for nephrin and Alexa Fluor™ 488 Goat anti-Guinea Pig IgG (H+L) Highly Cross-Adsorbed Secondary Antibody (A11073; Thermo Fisher Scientific) for Claudin-1 and Alexa Fluor™ 555 Donkey anti-rabbit IgG (H+L) Highly Cross-Adsorbed Secondary Antibody (A31572; Thermo Fisher Scientific) for Claudin-1 at 200x dilution, respectively.
